# Maternal high-fat-diet exposure is associated with elevated blood pressure and sustained increased leptin levels through epigenetic memory in offspring

**DOI:** 10.1038/s41598-020-79604-4

**Published:** 2021-01-11

**Authors:** Xian-Hua Lin, Ling Gao, Shen Tian, Christian Klausen, Meng-Xi Guo, Qian Gao, Miao-E. Liu, Hui Wang, Dan-Dan Wu, Cheng-Liang Zhou, Jing Yang, Ye Meng, Ye Liu, Gu-Feng Xu, Ya-Jing Tan, Kamran Ullah, Yi-Min Zhu, William D. Fraser, Jian-Zhong Sheng, Peter C. K. Leung, Louis J. Muglia, Yan-Ting Wu, He-Feng Huang

**Affiliations:** 1 Obstetrics and Gynecology Hospital, Institute of Reproduction and Development, Fudan University, Shanghai, China; 2 School of Medicine, The International Peace Maternity and Child Health Hospital, Shanghai Jiao Tong University, 910 Hengshan Road, Shanghai, 200030 China; 3 Shanghai Key Laboratory of Embryo Original Diseases, Shanghai, China; 4 The Key Laboratory of Reproductive Genetics, Ministry of Education, Zhejiang University, Hangzhou, China; 5 Department of Obstetrics and Gynecology, Child and Family Research Institute, University of British Columbia, Vancouver, BC V5Z 4H4 Canada; 6 Department of Pathology and Pathophysiology, School of Medicine, Zhejiang University, Hangzhou, China; 7 Department of Obstetrics and Gynecology, Maternity and Child Health Hospital of Songjiang District, Shanghai, China; 8 Centre de Recherche du Centre Hospitalier Universitaire de Sherbrooke (CRCHUS) and Department of Obstetrics and Gynecology, University of Sherbrooke, Sherbrooke, QC Canada; 9 Division of Human Genetics and Center for Prevention of Preterm Birth, Cincinnati Children’s Hospital Medical Center, Cincinnati, OH USA

**Keywords:** Developmental biology, Molecular biology, Health care

## Abstract

Maternal metabolism dysregulation during pregnancy predisposes offspring to major diseases, including hypertension, in later life, but the mechanism involved remains to be fully elucidated. A high-fat-diet (HFD) pregnant rat model was used to investigate whether excessive intrauterine lipid exposure was associated with elevated blood pressure in offspring and increased levels of leptin, an important biomarker and mediator of vascular dysfunction and hypertension. We found that gestational hyperlipidemia predisposed offspring to blood pressure elevation and sustained increases in leptin levels with no difference in body weight in the rat model. Increased *leptin* expression and *leptin* promoter hypomethylation were found in adipose tissues of HFD-exposed offspring. The treatment of mesenchymal stem cells with free fatty acids during adipogenic differentiation resulted in increased *leptin* expression, accompanied by *leptin* promoter hypomethylation. In addition, we also followed up 121 children to evaluate the association between maternal triglyceride levels and offspring blood pressure. Consistent with the animal study results, we observed elevated serum leptin levels and blood pressure in the offspring born to women with gestational hypertriglyceridemia. Our findings provide new insights that maternal hyperlipidemia is associated with elevated blood pressure in offspring and is associated with increases in leptin levels through epigenetic memory.

## Introduction

Hypertension is a leading risk factor for multiple life-threatening disorders, including coronary heart disease, cerebral events, and renal dysfunction, significantly contributing to the global burden of disease^1^. However, despite the availability of effective antihypertensives, the rate of the control of hypertension is far from satisfactory, which poses a need for the exploration of novel management strategies. Fetal programming is a notion that exposure to adverse intrauterine environments may increase the risk of adult disease in offspring^2,3^, and it is now recognized as a key determinant of the adult phenotype, with major implications for adult-onset diseases, including hypertension^4^. Thus, investigating the mechanisms underlying fetal programming of hypertension may provide novel clues for the management of hypertension.

Many maternal problems, including diabetes and obesity, affect fetal programming, especially hypertension^5,6^. Dyslipidemia is another equally prevalent metabolic disorder and is increasingly prevalent, with an overall rate > 40% in China^7^. Maternal malnutrition affects fetal programming and increases the risk of adult diseases, including hypertension, later in life^8–10^. Therefore, it is imperative to investigate the effect of maternal hyperlipidemia on blood pressure in offspring and potential mechanisms, which remain widely unknown.

Epigenetics provides a mechanistic link between environmental exposures and alterations in gene expression that might lead to healthy or unhealthy phenotypes^11^. To eliminate confounding factors and further explore the mechanism underlying maternal hyperlipidemia and hypertension in offspring, we first established a maternal high-fat-diet (HFD) pregnancy rat model to assess blood pressure and vascular function in offspring. We also examined leptin, an important biomarker and mediator of vascular dysfunction and hypertension, expression and methylation in adipose tissue and mesenchymal stem cells. Second, we conducted a follow-up study to investigate the blood pressure of the offspring born to mothers with different maternal triglyceride (TG) levels during pregnancy. Our findings will provide new insights into the development of hypertension and open up new possibilities for its prevention.

## Results

### Gestational hyperlipidemia was associated with elevation of blood pressure and mesenteric artery dysfunction in offspring

We established a maternal high-fat-diet pregnancy rat model to explore the association of gestational hyperlipidemia with blood pressure in offspring. The body weight and maternal serum lipid levels, including TGs, TC and LDL, were significantly higher in HFD-fed dams during pregnancy (Supplementary Fig. S1A–D), while HDL levels were lower in HFD rats (Supplementary Fig. S1E). No differences in serum glucose or insulin levels were observed (Supplementary Fig. S1F,G).

**Figure 1:**
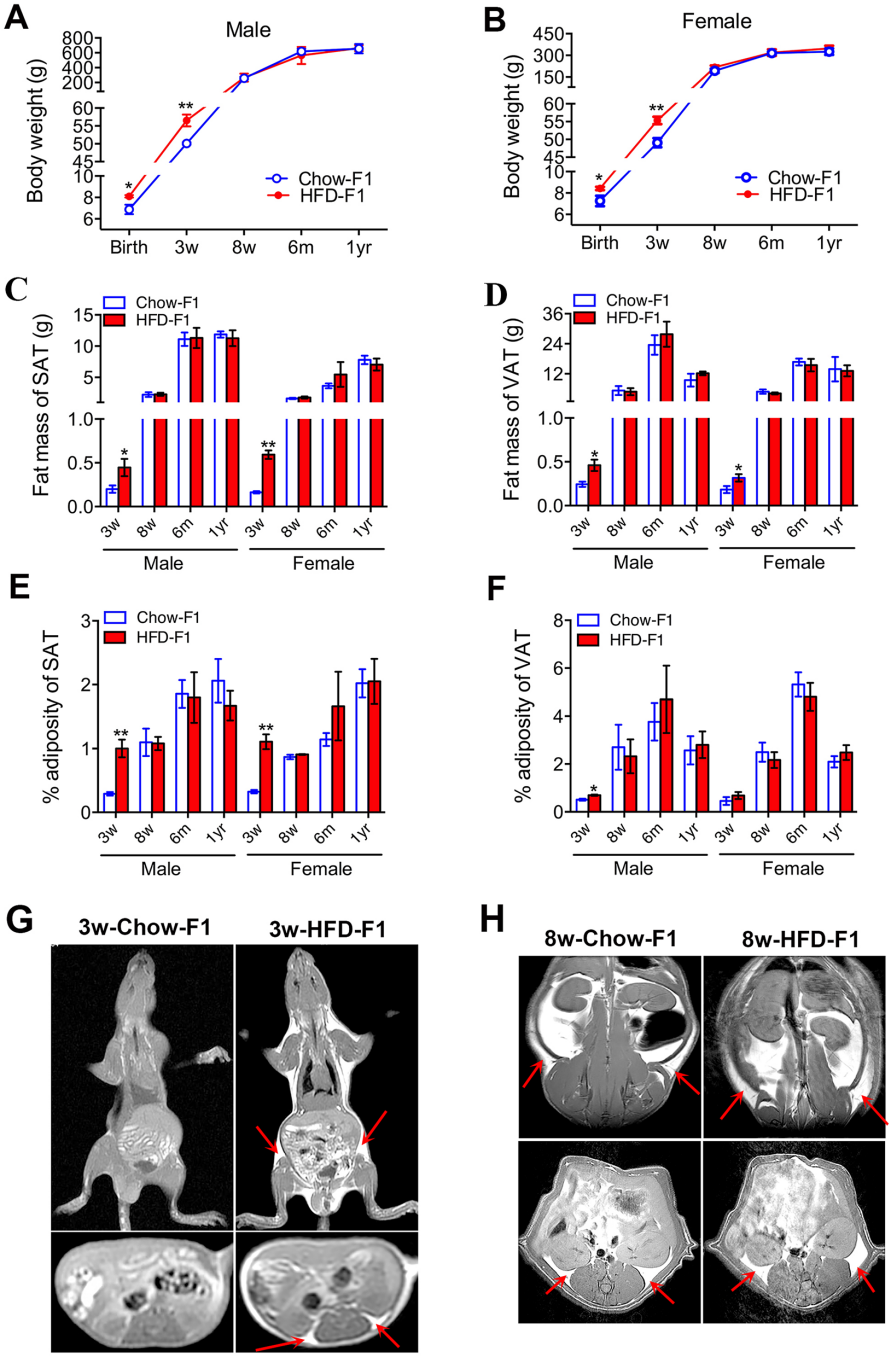
Body weight and fat mass between chow-exposed and HFD-exposed rat offspring at different ages. (**A**) Body weights of male chow-exposed and HFD-exposed rat offspring of various ages. (**B**) Body weights of female chow-exposed and HFD-exposed rat offspring of various ages. (**C**) Fat mass of subcutaneous adipose tissue (SAT) in male and female offspring. (**D**) Fat mass of visceral adipose tissue (VAT) in male and female offspring. (**E**) % adiposity (fat/body weight) of SAT in male and female offspring. (**F**) % adiposity of VAT in male and female offspring. (**G**) T1-weighted magnetic resonance (MR) images in 3-week-old chow-exposed and HFD-exposed male rat offspring. (**H**) T1-weighted MR images in 8-week-old chow-exposed and HFD-exposed male rat offspring. For (**A**–**F**), values are expressed as means ± SEs, n = 6 for each group. **p < 0.01, *p < 0.05, compared to the corresponding control. For E and F, the images above were obtained in the coronal plane; the images below were obtained in the transverse section. The arrows indicate fat distribution in subcutaneous and visceral adipose tissues.

In HFD-exposed pups, both males and females presented a higher body weight at birth and at 3 weeks of age (Fig. 1A,B). Fat masses of visceral and subcutaneous adipose tissues were increased in 3-week-old HFD-exposed pups regardless of sex (Fig. 1C–F). The results from magnetic resonance imaging (MRI) further confirmed the increased fat deposit in 3-week-old male pups born to HFD-fed dams (Fig. 1G). However, it was interesting to find that the differences in body weight and the percentage of adiposity were diminished between chow-exposed pups and HFD-exposed pups at 8 weeks after birth (Fig. 1C–F). MRI images in 8-week-old chow-exposed and HFD-exposed male pups presented no obvious difference in fat distribution in subcutaneous and visceral adipose tissues (Fig. 1H).

**Figure 2:**
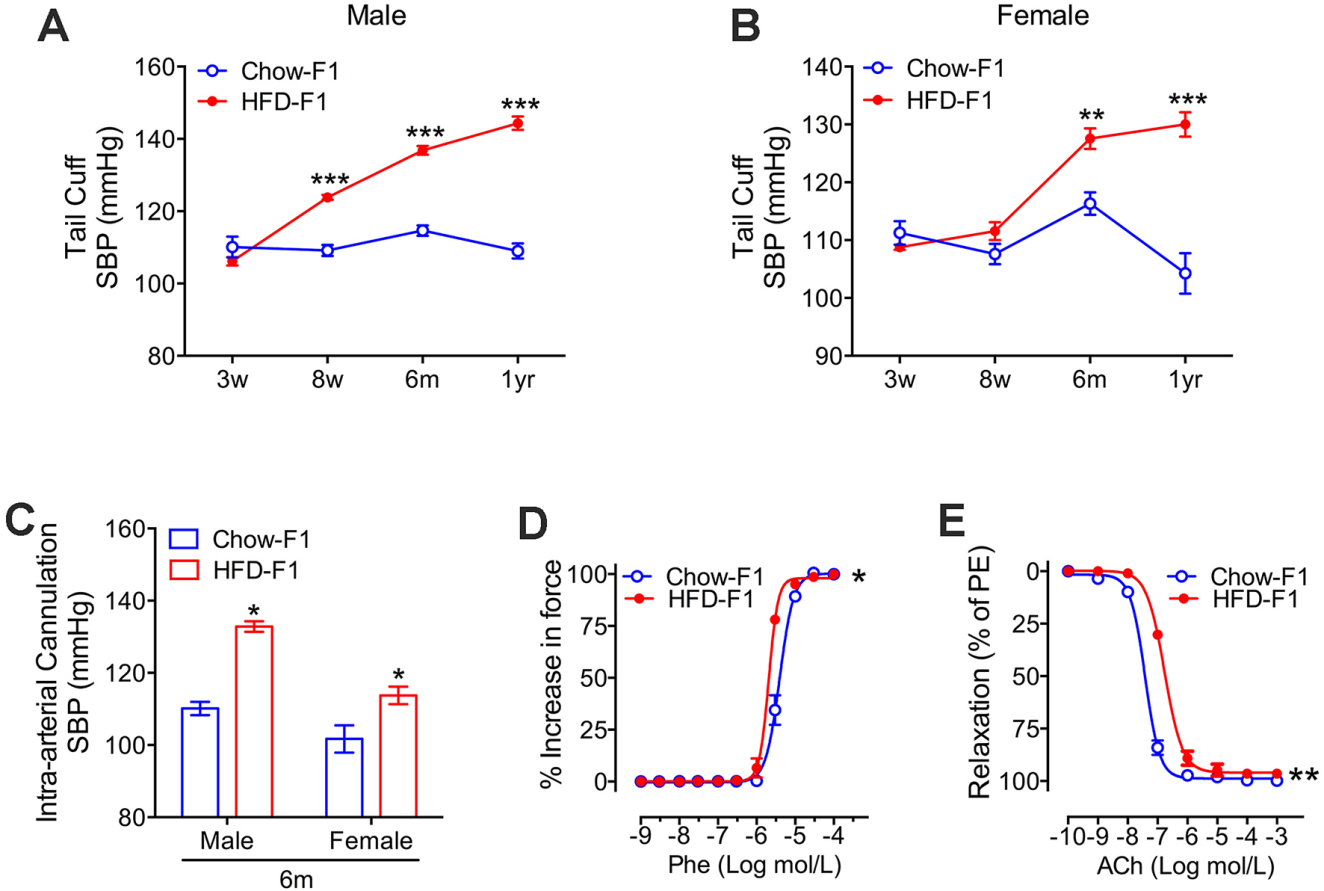
Maternal hyperlipidemia is associated with sustained elevated blood pressures, enhanced contraction, and reduced relaxation functions of the mesenteric arteries. (**A**) Tail cuff systolic blood pressures (SBP) in male chow-exposed and high-fat-diet (HFD)-exposed offspring (n = 6 for each group). (**B**) Tail cuff SBP in female chow-exposed and HFD-exposed offspring (n = 6 for each group). (**C**) Intra-arterial cannulation SBP in 6-month-old chow-exposed and high-fat-diet (HFD)-exposed offspring (n = 5 for each group). (**D**) The contractile responses of the mesenteric arteries to phenylephrine in one-year-old HFD-exposed and control male rat offspring (Phe, n = 3). The concentration for 50% of the maximal effect (EC_50_) in contraction is 3.973 × 10^–6^ M (Chow-F1) and 2.063 × 10^–6^ M (HFD-F1). (**E**) The endothelium-dependent vasorelaxation in the mesenteric arteries of one-year-old HFD-exposed and control male rat offspring (n = 3). The concentration for 50% of the maximal effect (EC_50_) in relaxation is 3.794 × 10^–8^ M (Chow-F1) and 1.705 × 10^–7^ M (HFD-F1). Values in (**A**,**B**) are expressed as means ± SEs, ***p < 0.0001 **p < 0.01, *p < 0.05, compared to the corresponding control.

Through non-invasive tail-cuff measurements, both male and female pups born to HFD-fed dams showed increases in systolic BP at 6 months and 1 year compared to control pups (Fig. 2A,B). Moreover, the increased blood pressure in pups born to HFD-fed dams was verified using invasive hemodynamic measurements in anesthetized rats (Fig. 2C). Furthermore, one-year-old HFD offspring presented an enhanced contractile response to phenylephrine (concentration for 50% of maximal effect (EC_50_) = 2.063 × 10^–6^ M in HFD rats vs. 3.973 × 10^–6^ M in controls, *p* < 0.05) and a reduced relaxation response to acetylcholine (EC_50_ = 1.705 × 10^–7^ M in HFD rats vs. 3.794 × 10^–8^ M in controls, *p* < 0.01) (Fig. 2D,E).

### Maternal hyperlipidemia was associated with increased leptin levels and reduction in leptin promoter methylation in adipose tissues

**Figure 3:**
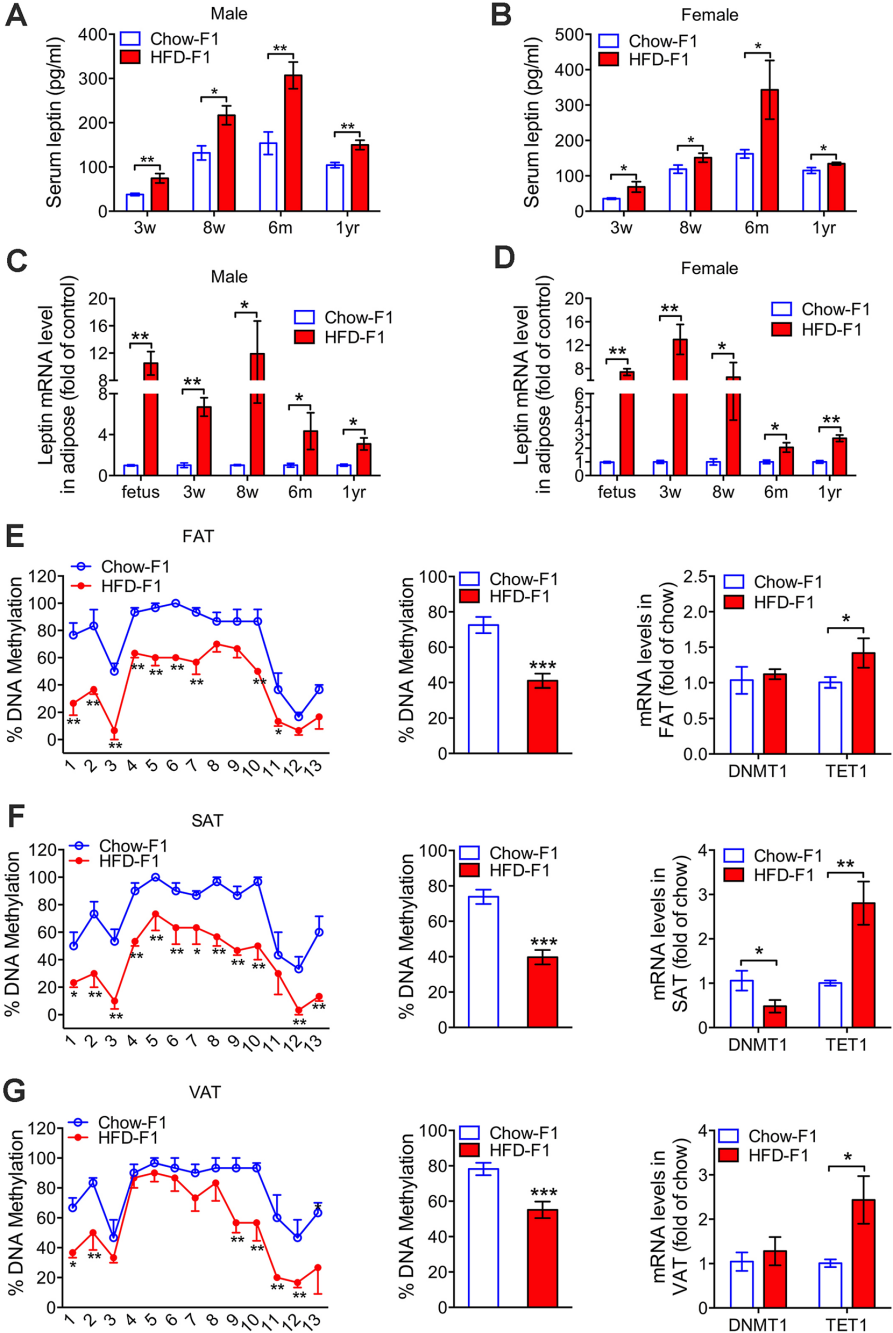
Long-term effect of gestational hyperlipidemia on *leptin* gene expression and methylation in adipose tissues of rat offspring. (**A**) Serum leptin levels in chow-exposed and HFD-exposed male rat offspring (n = 6 for each group). (**B**) Serum leptin levels in chow-exposed and HFD-exposed female rat offspring (n = 6 for each group). (**C**) Leptin mRNA levels in fetal adipose tissue (FAT) and adipose tissues (SAT and VAT) from chow-exposed and HFD-exposed male rat offspring at 3 and 8 weeks, 6 months and 1 year of age (n = 6 for each group). (**D**) Leptin mRNA levels in fetal adipose tissue (FAT) and adipose tissues (SAT and VAT) from chow-exposed and HFD-exposed female rat offspring at 3 and 8 weeks, 6 months and 1 year of age (n = 6 for each group). (**E**) The methylation levels of individual CpG sites (left, n = 3 for each group) and overall DNA methylation (middle) of the leptin promoter in FAT from male offspring; DNMT1 and TET1 mRNA levels in FAT (right, n = 4 for each group). (**F**) The methylation levels of individual CpG sites (left, n = 3 for each group) and overall DNA methylation (middle) of the leptin promoter in SAT from 3-week-old chow-exposed and HFD-exposed male rat offspring; DNMT1 and TET1 mRNA levels in SAT (right, n = 4 for each group). (**G**) The methylation levels of individual CpG sites (left, n = 3 for each group) and overall DNA methylation (middle) of the *leptin* promoter in VAT from 3-week-old chow-exposed and HFD-exposed male rat offspring; DNMT1 and TET1 mRNA levels in VAT (right, n = 4 for each group). Values are expressed as means ± SEs, ***p < 0.0001, **p < 0.01, *p < 0.05, compared to the corresponding control.

Leptin is well known to be an important biomarker and mediator of vascular dysfunction and hypertension^12,13^. Chronic administration of leptin increased blood pressure in experimental models^14,15^. To explore the mechanism linking maternal hyperlipidemia with elevated blood pressure in offspring, we examined the serum leptin levels in offspring at different ages in a rat model. Serum leptin levels were persistently higher both in HFD-exposed male and female pups than in chow-exposed pups at all ages (3 and 8 weeks, 6 months and 1 year; Fig. 3A,B). Moreover, we isolated fetal adipose tissue (FAT) and identified it by hematoxylin–eosin staining (Supplementary Fig. S2A–E). We observed that HFD-exposed pups displayed elevated leptin mRNA levels at all ages examined in adipose tissues in both male offspring and female offspring compared to their Chow-exposed counterparts (Fig. 3C,D). Elevated leptin protein levels in FAT from male HFD-exposed pups were confirmed by Western blot (Supplementary Fig. S3A). Likewise, immunohistochemical staining showed increased levels of leptin in the subcutaneous and visceral adipose tissues (SAT and VAT, respectively) of 3-week-old male pups and HFD-fed dams (Supplementary Fig. S3B).

Accumulating evidence suggests that altered epigenetic programming during embryonic and fetal development may explain how intrauterine nutrition exposure impacts health later in life^11^. Therefore, we analyzed the methylation of the *leptin* gene in adipose tissues from HFD-exposed and control male offspring. Importantly, quantitative DNA methylation analysis showed that the overall DNA methylation levels of the *leptin* gene were significantly reduced in the FAT (Fig. 3E), SAT (Fig. 3F) and VAT (Fig. 3G) of HFD offspring. Specifically, HFD-exposed offspring displayed significant hypomethylation of 9, 12 and 6 CpG sites in FAT, SAT and VAT, respectively (Fig. 3E–G). In addition, ten-eleven-translocation 1 (TET1, a maintenance DNA demethylase) mRNA levels were significantly increased in all adipose tissues (FAT, SAT and VAT; Fig. 3E–G) of offspring born to HFD-fed dams, while DNMT1 (an important maintenance methyltransferase) mRNA levels were reduced only in SAT.

### High levels of fatty acids reprogram leptin expression in mesenchymal stem cells (MSCs) during adipogenesis

**Figure 4:**
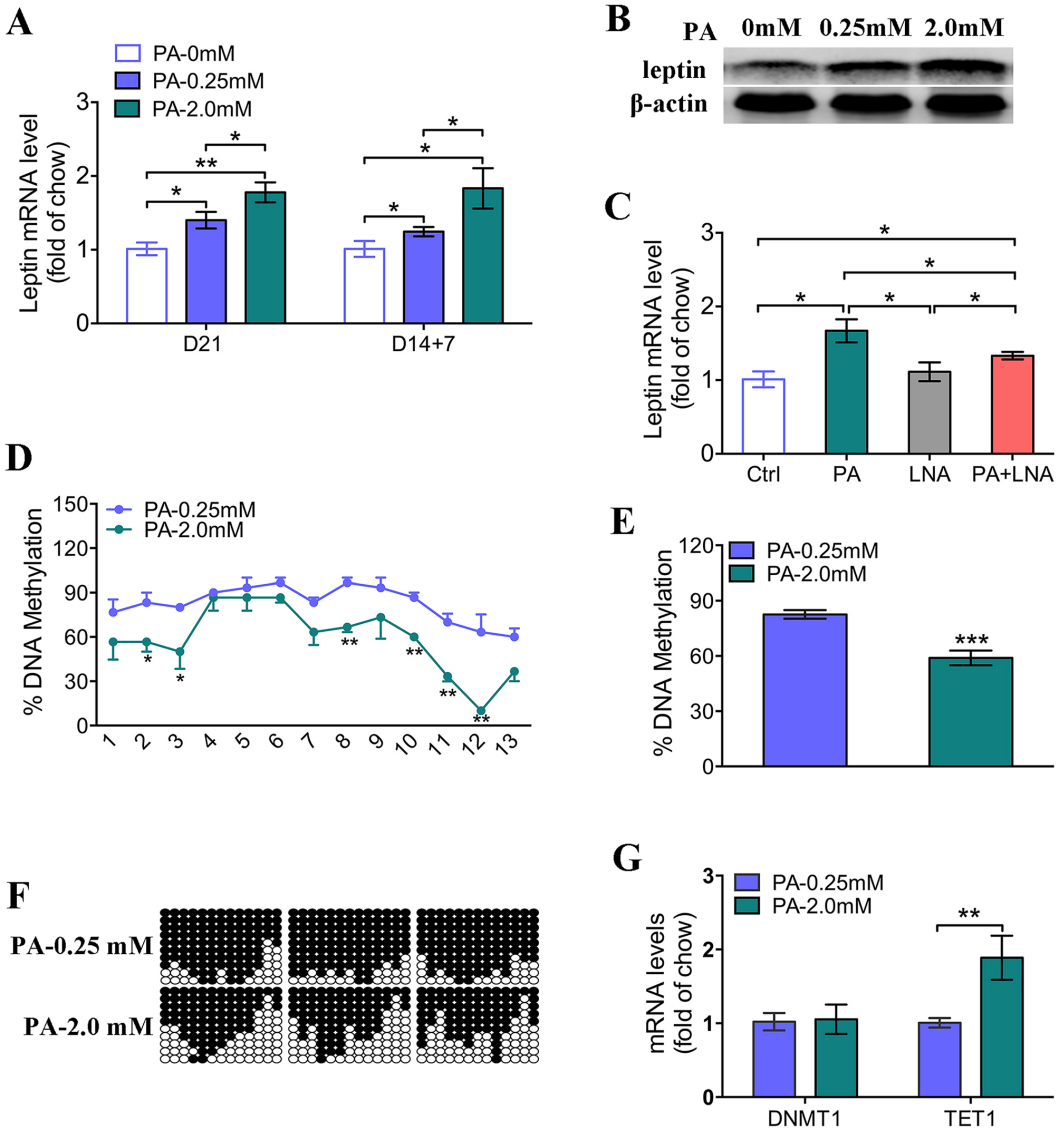
High levels of fatty acids reprogram leptin levels in rat mesenchymal stem cells (MSCs) during adipogenesis. (**A**) Leptin mRNA levels were measured following the treatment of MSCs for 21 days with vehicle or palmitic acid (PA; 0.25 or 2 mM; n = 4 for each group) or treatment with PA for 14 days followed by vehicle treatment for 7 days (D14 + 7). (**B**) Leptin protein levels were measured following the treatment of MSCs for 21 days with vehicle or PA. The grouping of gels cropped from different parts of the same gel. (**C**) Leptin mRNA levels were measured following the treatment of MSCs with vehicle, PA (1 mM) or PA (1 mM) plus linoleic acid (LNA; 1 mM) for 21 days. (**D**) Methylation levels of individual CpG sites within the leptin promoter in MSCs treated for 21 days with 0.25 or 2 mM PA (n = 3 for each group). (**E**) The overall methylation of the leptin promoter in MSCs treated for 21 days with 0.25 or 2 mM PA (n = 3 for each group). (**F**) The methylation profile of the leptin promoter in MSCs treated for 21 days with 0.25 or 2 mM PA (n = 3 for each group). (**G**) DNMT1 and TET1 mRNA levels were measured following the treatment of MSCs for 21 days with 0.25 or 2 mM PA (n = 4 for each group). Values are expressed as means ± SEs, ***p < 0.0001, **p < 0.01, *p < 0.05, compared to the corresponding control.

To determine whether the altered methylation profiles of *leptin* directly resulted from increased free fatty acid (FFA) levels during adipogenesis, we isolated MSCs from fetal rat bone marrow and treated them with palmitic acid (PA), a saturated fatty acid, during adipogenic differentiation. Successful isolation and adipogenic differentiation of MSCs were confirmed by transcription factor and surface marker expression analysis as well as Oil red O staining (Supplementary Fig. S4). The treatment of MSCs for 21 days with PA (0.25 or 2 mM) upregulated leptin mRNA and protein levels in a concentration-dependent manner (Fig. 4 A and 4B). Interestingly, the expression level of leptin at day 21 was persistently higher in the 2.0 mM group even after the withdrawal of PA treatment at day 14 during adipogenic differentiation (Fig. 4A). On the other hand, we treated mesenchymal stem cells (MSCs) with PA and linoleic acid (LNA) for 21 days. LNA is an unsaturated fatty acid that has been shown to prevent the effects of PA in vitro^16^. We found that treatment with LNA partially attenuated the effects of PA on leptin expression (Fig. 4 C). Compared to those treated with 0.25 mM PA, MSCs treated with 2 mM PA displayed reduced overall DNA methylation of the *leptin* promoter and hypomethylation of 6 CpG sites (Fig. 4D–F). Moreover, treatment of MSCs for 21 days with 2 mM PA significantly upregulated TET1 mRNA levels but did not alter those of DNMT1 (Fig. 4G). These findings suggest that FFAs directly contribute to the epigenetic reprogramming of leptin levels in rat MSCs during adipogenesis.

### High maternal triglyceride (mTG) levels were associated with elevated blood pressure and leptin levels in offspring

**Figure 5:**
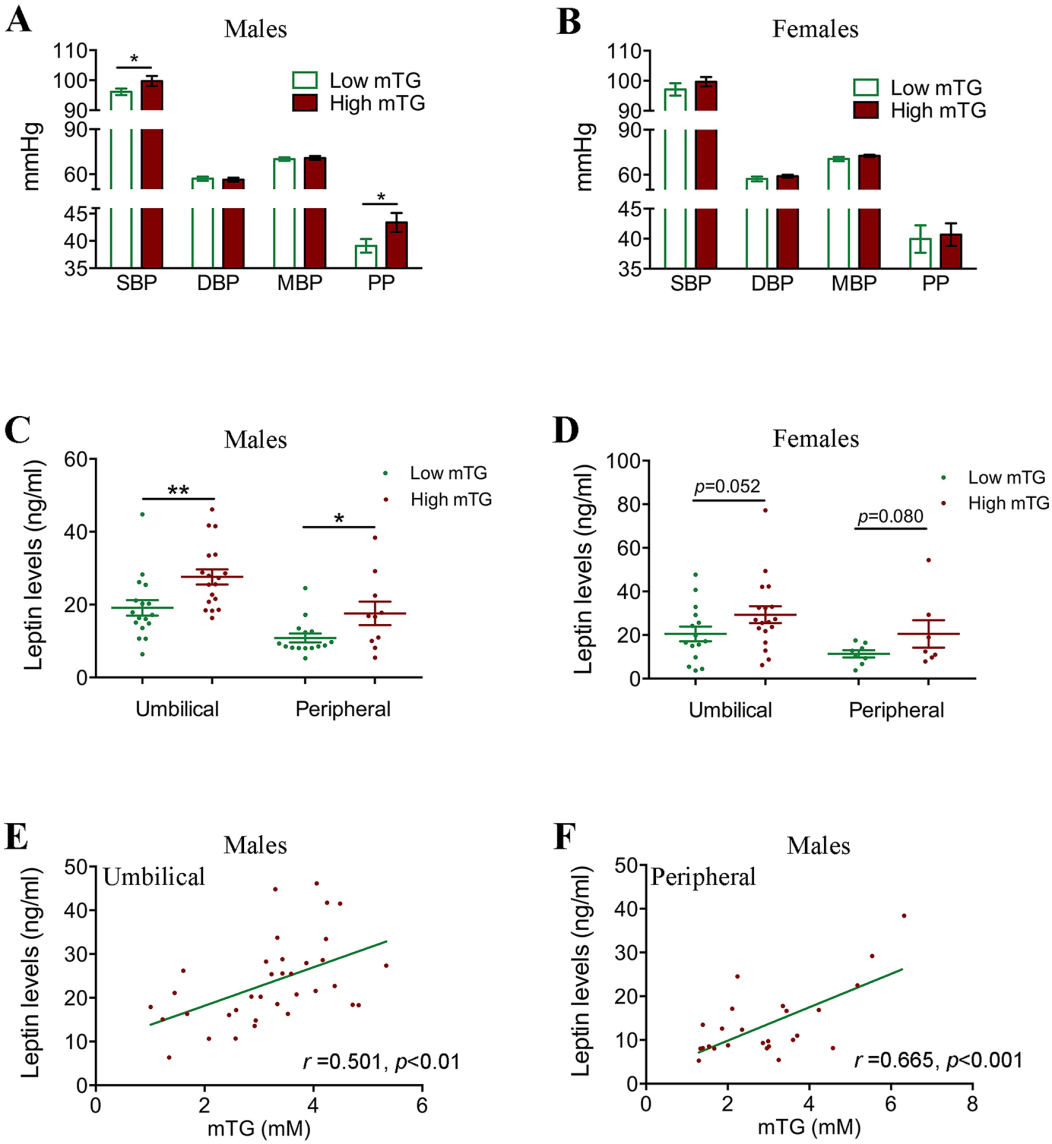
High maternal triglyceride (mTG) levels are associated with elevated blood pressure and leptin levels in offspring. (**A**) Blood pressures in male offspring of preschool-age children in the low- (n = 37) and high- (n = 36) mTG-level exposure groups. (**B**) Blood pressures in female offspring of preschool-age children in the low- (n = 22) and high- (n = 26) mTG-level exposure groups. (**C**) Serum leptin levels in male newborns and preschool-age children from low- (n = 17 and 15 for newborns and preschool-age children, respectively) and high- (n = 18 and 10 for newborns and preschool-age children, respectively) mTG-level mothers. (**D**) Serum leptin levels in female newborns and preschool-age children from low- (n = 1^5^ and 8 for newborns and preschool-age children, respectively) and high- (n = 18 and 7 for newborns and preschool-age children, respectively) mTG-level mothers. (**E**) Correlation between serum leptin levels in cord blood from male offspring and mTG levels (n = 35). (**F**) Correlation between male preschool-age children’s serum leptin levels and mTG levels (n = 25).

To further confirm whether hyperlipidemia was associated with elevated blood pressure, we carried out a follow-up study to investigate the blood pressure of 121 preschool-age children born to mothers with different mTG levels. Based on the mean mTG value from a large cohort of pregnant women (Supplementary Table S1), we categorized the mothers of these 121 children into high mTG (> 3.28 mM) or low mTG (≤ 3.28 mM) groups. Children born to mothers with high mTG levels showed significantly higher birth weight, while there was no difference in age, weight, height or BMI between the low-mTG-level group and the high-mTG-level group (Supplementary Table S2). Interestingly, the systolic pressures and pulse pressures tended to be significantly higher in male but not in female children born to mothers with high mTG levels (99.75 ± 9.93 vs. 96.13 ± 6.63; 43.38 ± 10.40 vs. 39.10 ± 7.70, respectively) (Fig. 5A,B). Moreover, we found significantly increased leptin levels in the high-mTG-level group both at birth and at preschool age in male offspring (Fig. 5C), even though their BMIs and BMI *z*-scores did not differ at preschool age (Supplementary Table S2). No significantly increased leptin levels were found in female offspring (Fig. 5D). We then conducted an analysis of clinical data to explore the potential association between mTG levels and leptin levels in male newborns and preschool-age children. We found that leptin levels were positively correlated with mTG levels both in newborns and preschool children (Fig. 5E,F).

Considering the higher incidence of macrosomia (birth weight ≥ 4000 g) in pregnant women with high TG levels (15.82% vs. 8.86%; Supplementary Table S1), we further investigated the blood pressure of 1824 children, including 858 preschool-age children (3–6 years old), 966 school-age children (7–14 years old) and 3079 adults (18–50 years old), with different birth weights. Not surprisingly, maternal TG levels were higher in the macrosomia group than in the control group (birth weight, 2500–3999 g; Supplementary Fig. S5A; Supplementary Table S3). The mean systolic and diastolic pressures were significantly higher in adults who were macrosomic at birth than that in controls (Supplementary Fig. S5B; Supplementary Tables S5–S6). The incidence of hypertension in adults (30–50 years) was significantly higher in the macrosomia group than in the control group (32.46% vs. 14.98%; Supplementary Fig. S5C and Supplementary Table S7). Moreover, we found that serum leptin levels were persistently higher in the macrosomia group at all ages (newborn, preschool, school and adult), even though their BMIs did not differ at school age or in adulthood (Supplementary Fig. S5D).

### Reduced leptin promoter methylation was confirmed in newborn and preschool-age children born to high-mTG-level mothers

**Figure 6:**
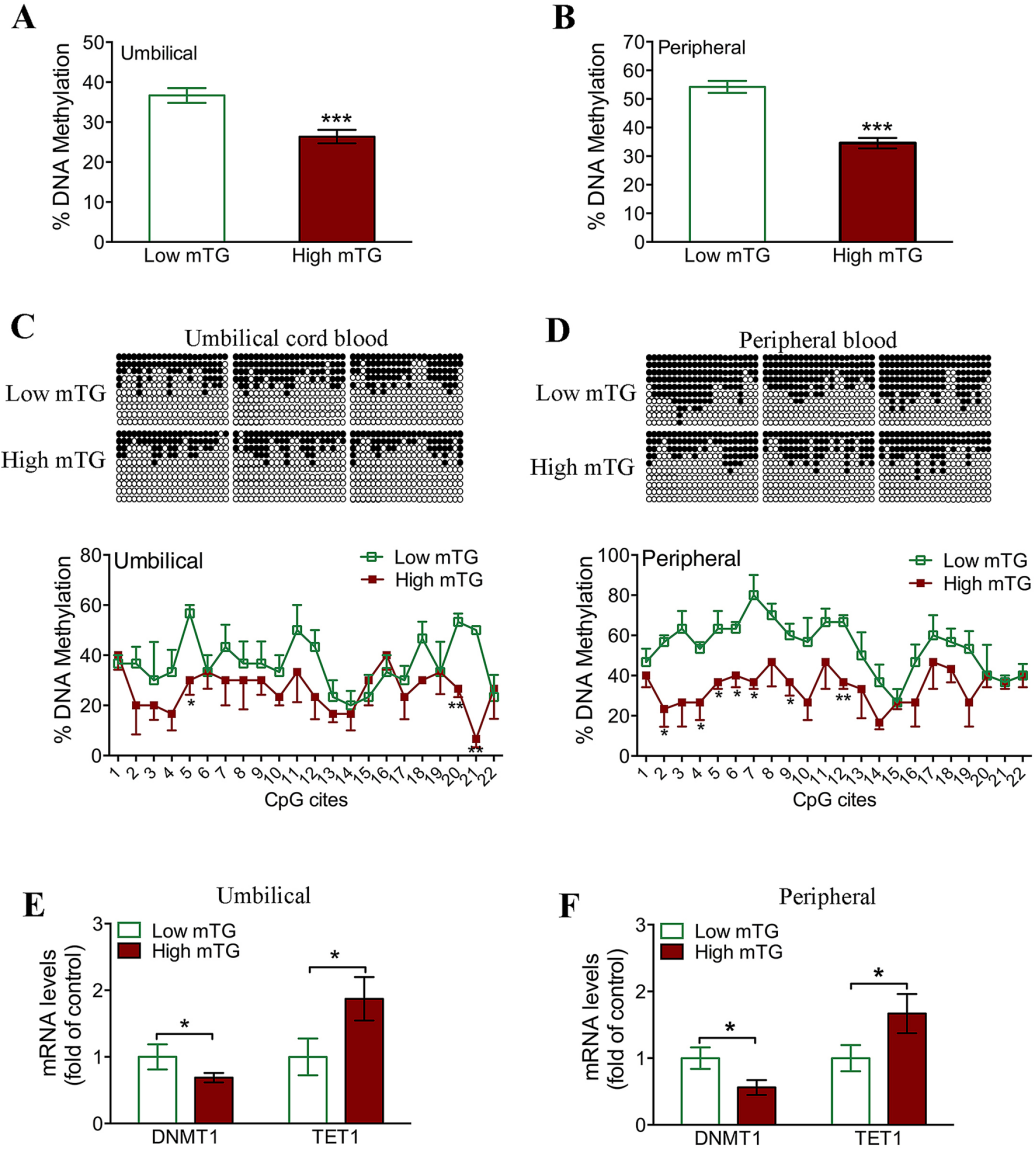
Reduced leptin promoter methylation was confirmed in newborn and preschool-age children born to high-mTG-level mothers. (**A**) The overall methylation of the leptin promoter in male umbilical cord blood lymphocytes from the low- and high-mTG-level groups (n = 3 for each group). (**B**) The overall methylation of the leptin promoter in lymphocytes of male preschool-age children from the low- and high-mTG-level groups (n = 3 for each group). (**C**) Bisulfite sequencing was used to measure methylation levels of individual CpG sites within the leptin promoter in umbilical cord blood lymphocytes from the low- and high-mTG-level groups. (**D**) Methylation levels of individual CpG sites within the leptin promoter in lymphocytes of preschool-age children from the low- and high-mTG-level groups. (**E**) DNMT1 and TET1 mRNA levels in umbilical cord blood lymphocytes from the low- and high-mTG-level groups (n = 12 for each group). (**F**) DNMT1 and TET1 mRNA levels in lymphocytes of preschool-aged children from the low- and high-mTG-level groups (n = 12 for each group). Values are expressed as means ± SEs, ***p < 0.0001, **p < 0.01, *p < 0.05, compared to the corresponding control.

Next, we addressed whether *leptin* gene methylation is truly altered in the offspring of women with gestational hypertriglyceridemia. Consistent with our findings in rats, bisulfite sequencing revealed that overall DNA methylation of the leptin promoter in lymphocytes was significantly reduced in male newborn (Fig. 6A) and preschool-age children (Fig. 6B) born to high-mTG-level mothers. Moreover, male newborns in the high-mTG-level group exhibited significant hypomethylation at 3 of 22 CpG sites (Fig. 6C), while male preschool-age offspring exhibited hypomethylation of 7 CpG sites (Fig. 6D). Interestingly, we found the upregulation of the demethylase TET1 as well as the downregulation of DNMT1 at both ages in the high-mTG-level group (Fig. 6E,F).

## Discussion

In this study, we established a rat model of maternal hyperlipidemia and found that excessive intrauterine lipid exposure was associated with elevated blood pressure in offspring, which was associated with a sustained increase in serum leptin levels in offspring. To substantiate these findings, we conducted a clinical investigation and found that preschool-age children born to mothers with high mTG levels presented higher systolic pressures and pulse pressures, especially in male individuals. Serum leptin levels were increased in the offspring of women with hypertriglyceridemia during pregnancy, and were positively correlated with mTG levels. Thus, our results suggest that maternal hyperlipidemia during pregnancy might be associated with an elevation of blood pressure and chronically elevated leptin levels in offspring.

A growing number of studies have shown that abnormal perinatal lipid levels are independently associated with cardiovascular and metabolic disease in offspring^17,18^. Gestational hypercholesterolemia has been suggested to predispose offspring to inflammation and cardiovascular risk in adult life^19,20^. Exposure to an HFD in utero has been shown to generate metabolic abnormalities in offspring. Here, our findings indicated that high mTG levels were associated with arterial dysfunction in adulthood. In the clinical investigation, we also found that preschool-age children born to mothers with high mTG levels presented higher systolic pressures and pulse pressures.

Leptin is a key contributor to elevation of blood pressure. Simonds et al. demonstrated that the increased leptin levels observed in mice with diet-induced obesity induced an increase in blood pressure^21^. Moreover, elevated serum leptin levels were also observed in intrauterine growth-restricted rat offspring and subsequently contributed to increased blood pressure later in life^22^. In current study, we observed that the rat offspring born to maternal hyperlipidemia exhibited elevated leptin levels and had higher blood pressure in adults, even though there was no difference in their BMI. High leptin can increase blood pressure via the pathway of the neurotransmitter glutamate^23^. Leptin is also thought to contribute to the pathogenesis of hypertension by stimulating vascular inflammation, oxidative stress, and vascular smooth muscle dysfunction^24–26^. Indeed, our studies showed enhanced contraction and reduced relaxation functions of the mesenteric artery in HFD-exposed offspring.

A variety of environmental factors (including dietary components) have been shown to influence gene expression by altering methylation patterns in CpG islands within promoter regions^27,28^. Our data indicate that increased leptin levels and *leptin* promoter hypomethylation were present in adipose tissues from rat offspring born to maternal hyperlipidemia. Similar results were found in rat lymphocytes (data not shown). Considering the limited access to adipose from human participants and the consistency of adipose and lymphocyte results in animal models, we further examined the leptin levels and leptin methylation in lymphocytes of children born to mothers with high TG levels, and the observed results were consistent with animal findings. Ten-eleven-translocation 1 (TET1) has been shown to play important roles in reprogramming genes in MSCs, adipocytes and peripheral blood lymphocytes^29^. We observed increased expression of TET1 coupled with reduced expression of DNMT1 in lymphocytes from children with mothers with high TG levels. Moreover, we investigated whether FFAs affected leptin expression and TET1 expression in PA-treated rat MSCs during adipogenic differentiation, to further substantiate the reprogramming of leptin in adipose tissue by intrauterine hyperlipidemia. TGs are key mediators in fetal growth^30^, and TGs can be broken down to yield FFAs, which have been shown to modulate the expression of adipogenic transcription factors in 3T3-L1 adipocytes^31^. Our results indicated elevated leptin levels in conjunction with reduced *leptin* promoter methylation in PA-treated MSCs, suggesting a mechanism whereby high mTG levels can reprogram leptin levels in offspring. Importantly, the effect of PA on the expression and methylation of leptin was partially rescued by LNA, an unsaturated fatty acid that has been shown to prevent the effects of PA in vitro^16^, which might illuminate a possible target for the prevention of fetal origins of adult hypertension.

In addition, we observed that males are more sensitive to maternal malnutrition and that a more robust response in male offspring than in female offspring accounts for the alterations in blood pressure and leptin expression, which is similar to other studies^32,33^. Although the mechanisms responsible for these alterations were not the focus of the present studies, future investigations will focus on elucidating the potential mechanisms by which exposure to an HFD in utero and postnatally alters the cardiovascular system.

In summary, we clearly demonstrate that intrauterine exposure to hyperlipidemia is associated with elevated blood pressure in adulthood, which is related to sustained increase in serum leptin levels via epigenetic reprogramming of the leptin promoter. Our findings underscore the importance of balancing maternal lipid metabolism during pregnancy and may help in the development of strategies aiming at reducing or preventing hypertension related to fetal exposure to intrauterine hyperlipidemia.

## Materials and methods

### Animals and experimental model

After acclimatization for 2 weeks, eight-week-old Sprague–Dawley (SD) rats were randomly assigned to two groups: a normal control diet (chow, n = 20) or a high-fat diet (HFD: 18.7% lard, 11.4% sucrose, 12% casein, 2.5% maltodextrin, 1.3% cholesterol, 0.3% cholate and 52% standard chow, n = 20). Virgin female SD rats were mated with normal male SD rats at 10 weeks, and pregnant females were fed chow or an HFD from 8 weeks to delivery. Body weight and lipid levels were measured every week from 8 weeks to delivery. Pregnancy onset was assessed by the presence of a copulation plug after overnight mating (designated as day 0 (D0) of pregnancy). Maternal tail blood lipid levels were measured on D7, D14, and D20 of pregnancy. Twenty dams fed an HFD were used to generate rat pups. Five litters were used for the collection of MSCs during pregnancy. Fifteen litters of pups were enrolled to investigate the phenotype and study mechanism involved. At every time point, one pup per litter of each sex was enrolled in the study, and the ratio of males to females was 1:1 in every group. The litter size was randomly reduced to ten after delivery to ensure uniformity. Pups from HFD-fed mothers were fostered by control females until they were weaned. After weaning, the HFD-exposed offspring were given the same diet as the controls. Animal care and management procedures were in accordance with the institutional guidelines for laboratory animals established by the Animal Care and Use Committee (ACUC) and were approved by the ACUC, School of Medicine, Zhejiang University, Hangzhou, China.

### Magnetic resonance imaging (MRI)

Subcutaneous adipose tissue and visceral adipose tissue were determined by MRI. Male rats were imaged on a GE Signa HDXT system 1.5-T scanner with a 5-inch surface coil and a T1-weighted fast-spin echo pulse sequence (repetition time msec/echo time msec, 540/9.0; number of excitations, 6; field of view, 20 × 20 cm; matrix size, 320 × 224). The whole chest and abdomen of each rat were covered with axial slices and coronal slices (2-mm thick, no spacing). All images acquired were retrieved from the MR scanner and transferred to personal computers in DICOM (Digital Imaging and Communication In Medicine) format.

### Blood pressure measurement and tissue preparation

The rat offspring underwent a systolic BP test and orbital blood collection at 3 weeks, 8 weeks, 6 months and 1 year of age. Systolic BP was measured by the tail-cuff technique in conscious animals with a noninvasive automatic sphygmomanometer (BP98A, Softron, Tokyo, Japan) at the location of the tail artery while the animals were restrained and placed on a temperature-controlled pad after a 30-min stabilization period. Rats were acclimated to the tail-cuff apparatus at least twice. The BP was monitored for 15 min under a restrained condition, and ten readings were taken for each measurement. The average value was calculated and determined. Serum and lymphocytes were separated immediately after collection and stored at -20 °C for later analysis. Invasive hemodynamic measurements in anesthetized rats from a new cohort were performed for all groups of offspring (n = 10 rats/group) with a 2 F pressure catheter inserted into the right carotid artery and guided into the left ventricle. BP was continuously recorded with a PowerLab data-acquisition system (LabChart version 8.1.16, AD Instruments).

Visceral fat (gonadal, retroperitoneal, and perirenal) and subcutaneous fat (inguinal) were collected under anesthesia (chloral hydrate 0.04 g/kg, ip) after 12 h of fasting and weighed. Fetal adipose tissue was extracted from fetuses subcutaneously within 24 h of birth and embedded in paraffin or processed for RNA, DNA or protein extraction as described below.

### Wire myograph and the measurement of vascular responses

The experiment was performed in one-year-old male offspring. Small-vessel myography was performed as previously described in detail^34^. In brief, after one-year-old rat offspring were sacrificed by decapitation, mesenteric arteries (third-order branch of the superior mesenteric artery) were isolated and cut into 2 mm rings in ice-cold PBS solution and mounted on a Mulvany-Halpern wire myograph (model 620 M, Danish Myo Technology, Aarhus, Denmark). Following 30 min of equilibration, the vessels were normalized according to a standardized procedure. The vessels were routinely allowed to equilibrate for an additional 1 h before the start of the experiments. Arterial rings were precontracted with phenylephrine (Phe, 3 μM) and subsequently relaxed with the cumulative application of acetylcholine (ACh) in increments from 10^–10^ to 10^–3^ M. Then, we performed the second experiment to determine the contractile responses of segments of the mesenteric arteries to Phe (cumulative concentration from 10^–9^ to 10^–4^ M).

### Serum lipid analysis

Serum concentrations of triglycerides, total cholesterol (TC), low-density lipoprotein (LDL) and high-density lipoprotein (HDL) were measured (Architect C1600; Abbott Laboratories, Abbott Park, IL, USA) in the clinical chemistry laboratory of Women’s Hospital, School of Medicine, Zhejiang University. Serum leptin levels were quantified with a human and rat enzyme-linked immunosorbent assay kit (XiTang Bio Technology Co, Shanghai, China) with a detection sensitivity of 0.3 ng/mL and intra- and interassay coefficients of variation of less than 10%.

### DNA extraction, bisulfite conversion and sequencing

Genomic DNA was extracted from lymphocytes with the TIANamp genomic DNA kit (Tiangen Biotech, Beijing, China). A partial human *leptin* promoter sequence spanning –2928 to + 23 with respect to the transcription start site (GenBank U43589) was used to design bisulfite sequencing PCR primers with MethMrimer tools (http://www.urogene.org/methprimer/, UCSF), and 22 potential CpG sites were identified in a region spanning − 496 to − 304. Previous studies have identified 13 CpG sites in a region spanning − 694 to − 372 in the promoter of the rat *leptin* gene^35^. Bisulfite conversion was performed with the Methylamp DNA Modification Kit (Qiagen, Hillden, Germany). Three samples from each group were randomly selected for *leptin* promoter methylation analysis by bisulfite sequencing of 10 clones per sample. Global methylation levels as well as the methylation levels of individual CpG sites were calculated.

### BM-MSC isolation, culture and adipogenic differentiation

Pregnant SD rats were anesthetized on day 13 of gestation, and MSCs were isolated from fetal bone marrow and expanded as previously described^36^. MSCs were treated with 0.5 mM 1-methyl-3 isobutylxanthine, 1 μM dexamethasone and 10 μg/mL insulin in the absence or presence of palmitic acid (Sigma, St Louis, USA), linoleic acid (Sigma, St Louis, USA) or palmitic acid plus linoleic acid for 3 weeks. Adipogenic differentiation was confirmed through the analysis of transcription factor expression by reverse transcription PCR and surface expression of MSC markers (positive: CD44 and CD90; negative: CD45; all from eBioscience, San Diego, USA) by flow cytometry (Cytomics FC500, Beckman Coulter, CA, USA). Lipid droplet formation was confirmed in formalin-fixed cells with 60% isopropanol washes and Oil Red O staining.

### Western blotting and immunohistochemical analyses

Western blot analysis was performed as described previously^37^. Briefly, samples were separated by SDS-PAGE, transferred to polyvinylidene difluoride membranes and incubated overnight at 4 °C with rabbit polyclonal anti-OB (1:1000; Millipore), anti-PPARγ (1:1000; Proteintech), anti-FABP4 (1:500; Proteintech) or mouse polyclonal anti-β-actin (1:10,000; Abcam) antibodies. Membranes were then incubated for 1 h at room temperature with fluorescence-labeled anti-mouse or anti-rabbit IgGs (1:5000) prior to visualization with an Odyssey Imager (LI-COR, Odyssey, NE, USA). Formalin-fixed adipose tissues were sectioned at 4 μm intervals, blocked with 1% BSA, and incubated overnight at 4 °C with anti-OB, anti-PPARγ and anti-PABP4 (1:1000 dilution for each antibody).

### Study population

Third-trimester serum triglyceride levels were analyzed in 2687 Han Chinese women who delivered singleton babies at the Women’s Hospital, School of Medicine, Zhejiang University between January 2006 and December 2012. Women with hypertensive and diabetic diseases (including gestational diabetes), intrahepatic cholestasis of pregnancy, placental abruption and placenta previa, multifetal gestations, preterm delivery, or post-term pregnancy were excluded. Among these women, we followed up 121 children aged 3–6 years old and collected 68 umbilical cord blood samples and 40 peripheral blood samples to examine serum leptin levels. We also performed a retrospective cohort study and recruited an additional 4782 Han Chinese people with birth weight records, including 1703 children and 3079 adults, from the Child Care Center and the Physical Examination Center of Hangzhou First People's Hospital, Hangzhou, China. Participants from the retrospective cohort (n = 4782) and 121 children aged 3–6 years old from the first cohort (total n = 4903) were first divided into a macrosomia group (≥ 4000 g) and a normal birth weight control group (2500–3999 g) and then further subdivided into preschool-age (3–6 years, n = 858), school-age (7–14 years, n = 966), and adult (18–50 years, n = 3079) groups. We also collected 55 blood samples (32 samples from school-age children and 23 samples from adults) after 12 h of fasting for leptin analysis. Children born preterm, small for gestational age or with nephritis, nephrosis or other diseases leading to secondary hypertension were excluded. BMI and blood pressure (BP) were measured in all subjects. BP was manually measured with standard sphygmomanometers, and Korotkoff sounds (phase V) were used for estimations of diastolic BP (DBP). Pulse pressure (PP) was defined as the numeric difference between systolic blood pressure and diastolic blood pressure. Subjects were required to rest for more than 5 min in a seated position before BP measurement. Three BP measurements were performed one minute apart. The mean of the second and third measurements was recorded. The study was approved by the Research and Ethics Committee of the University Hospital of China and was registered in the Chinese Clinical Trial Registry (ChicCTR-OCH-14004536, www.medresman.org). Informed consent was obtained from all adult subjects and the parents of each child. In addition, we also confirmed that all methods were performed in accordance with the relevant guidelines and regulations.

### Statistical analyses

All data were normally distributed and expressed as means ± SDs or SEMs. The independent-sample *t* test, nonparametric test, and chi-squared tests were used to evaluate the statistical significance between two groups. One-way ANOVA and Tukey’s post hoc tests were used to evaluate the statistical significance of the difference between more than 2 groups. Statistical analyses were performed with SPSS version 19.0 for Windows. *P* values < 0.05 were considered statistically significant.

## Supplementary information

Supplementary Information.
