# Supplementary material for: Maternal high-fat-diet exposure is associated with elevated blood pressure and sustained increased leptin levels through epigenetic memory in offspring
Source: Sci Rep. 2021 Jan 11;11:316. doi: 10.1038/s41598-020-79604-4 (PMC7801715; doi:10.1038/s41598-020-79604-4)
Supplement: Supplementary file 1 — Supplementary Information. [file 41598_2020_79604_MOESM1_ESM.pdf]

Supplementary Information for

Maternal high-fat-diet exposure is associated with elevated blood pressure and sustained increased leptin levels through epigenetic memory in offspring

Xian-Hua Lin[1,3#], Ling Gao[1#], Shen Tian[4#], Christian Klausen[5], Meng-Xi Guo[2,3,] , Qian Gao[4,6], Miao-E Liu[4], Hui Wang[7], Dan-Dan Wu[2,3], Cheng-Liang Zhou[2,3], Jing Yang[4], Ye Meng[4], Ye Liu[2,3], Gu-Feng Xu[4], Ya-Jing Tan[2,3], Kamran Ullah[4], Yi-Min Zhu[4], William D. Fraser[8], Jian-Zhong Sheng[4,6], Peter C.K. Leung[5], Louis J. Muglia[2,9], Yan-Ting Wu[1,3*], He-Feng Huang[1,2,3,4*]

Corresponding author: He-Feng Huang and Yan-Ting Wu

E-mail: huanghefg@hotmail.com, and yanting_wu@163.com

Supplementary Figure 1. Baseline characteristics of pregnant rats in control and HFD rats (F0).

Fig. S1

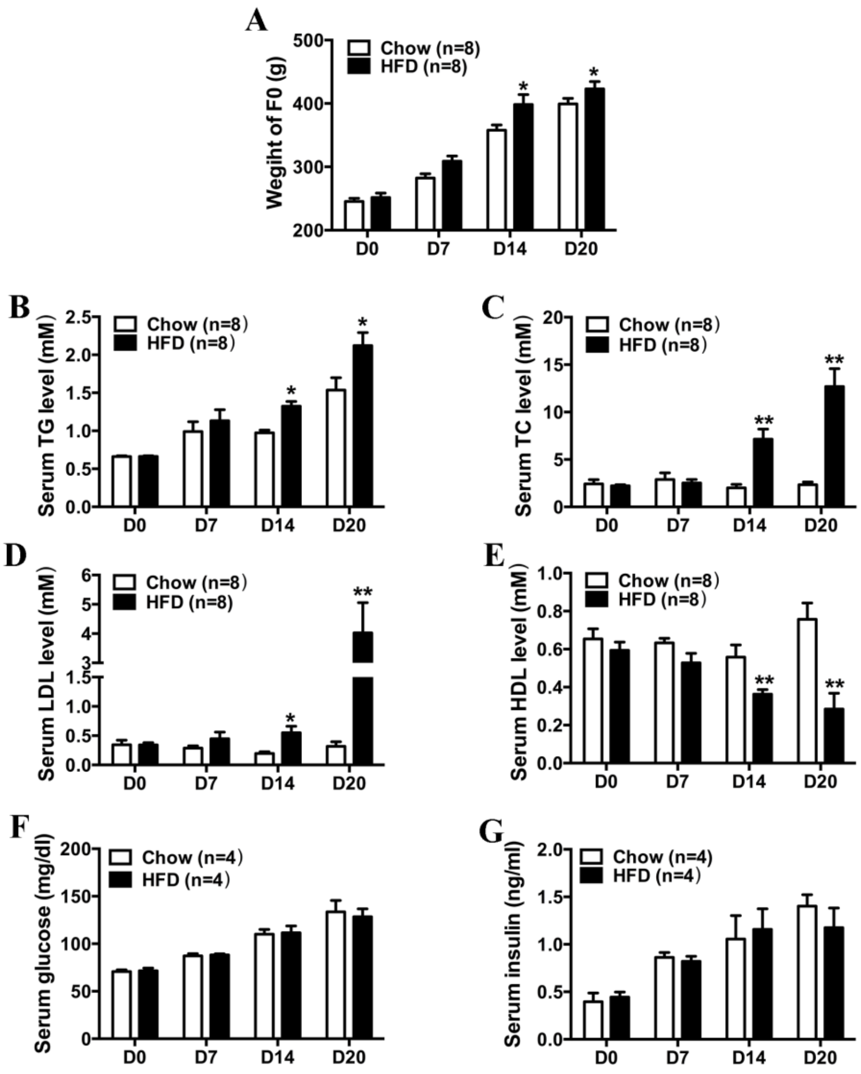

Fig. S1. Baseline Characteristics of Pregnant Rats in Control and HFD Rats (F0). (A) Body weights before pregnancy (mating day, D0) and during pregnancy (D7, D14, D20) in chow and HFD female rats. (B, C, D and E) Serum levels of lipids before and during pregnancy in chow and HFD female rats. (F and G) Serum glucose and insulin levels before and during pregnancy in chow and HFD female rats. Values are expressed as means ± SE, *P<0.05, **P<0.01, compared to the corresponding control.

Supplementary Figure 2. Identification of fetal adipose tissue (FAT).

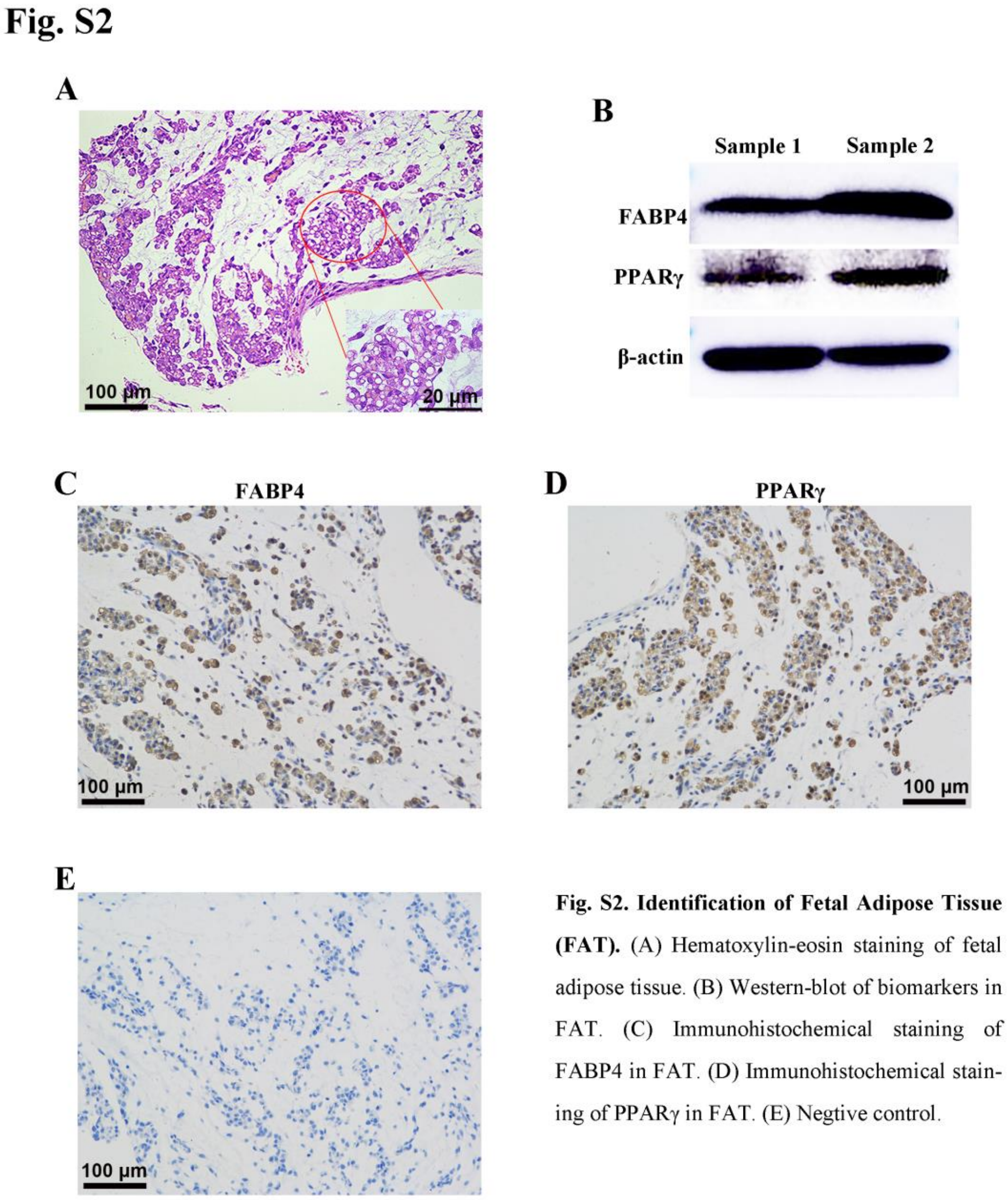

Supplementary Figure 3. Leptin expression in adipose tissues between Chow-F1 and HFD-f1 groups.

Figure S3

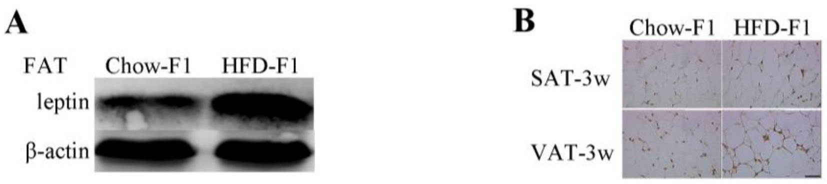

Fig. S3 Leptin expression in adipose tissues between chow-exposed and HFD-exposed groups. (A) Leptin protein levels in FAT from chow-exposed and HFD-exposed rat offspring. The grouping of gels cropped from different parts of the same gel. (B) Immunohistochemical staining of leptin in subcutaneous and visceral adipose tissues (SAT and VAT, respectively) from 3-week-old chow-exposed and HFD-exposed rat offspring (×400, Scale bars: 50 μm).

Supplementary Figure 4. Identification of MSCs isolated from fetal rat (D13) bone marrow.

Fig. S4

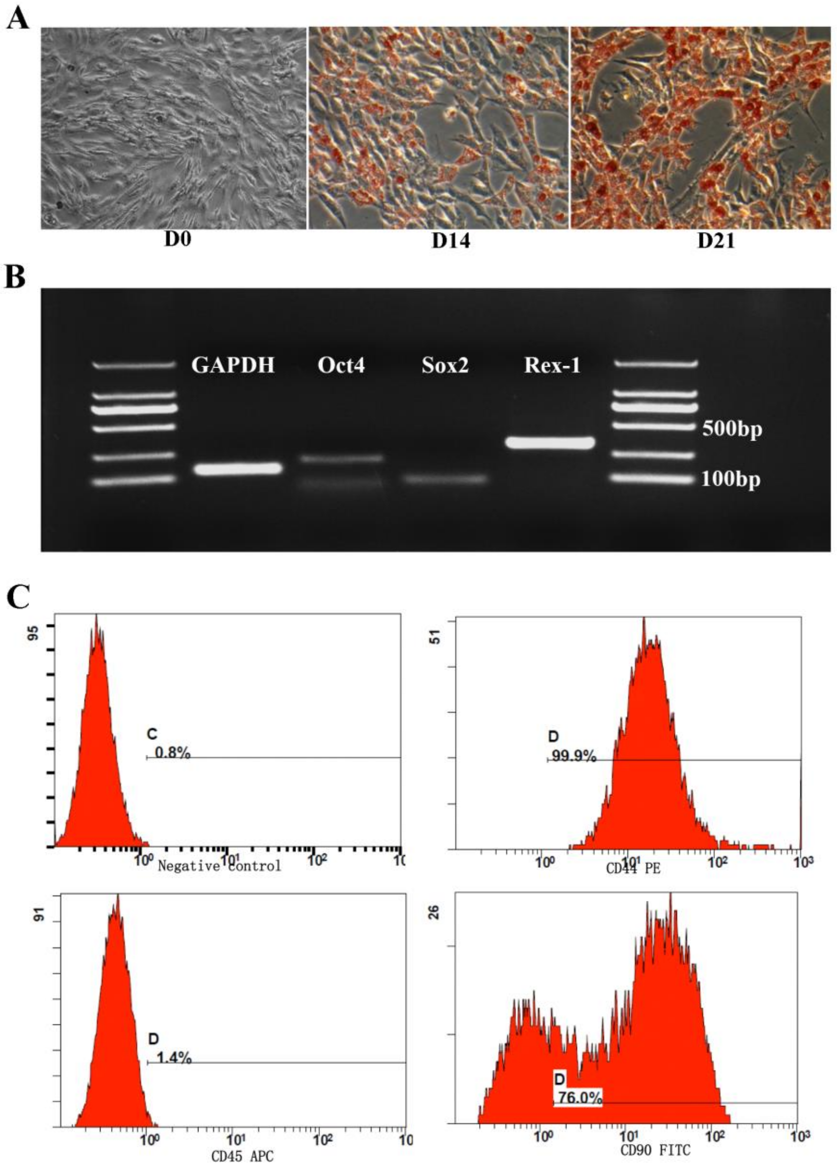

Fig. S4 Identification of MSCs Isolated from Fetal Rat (D13) Bone Marrow. (A) Morphology of MSCs before differentiation (D0), Oil red staining at days 14 and 21 of proliferation. (B) Determination of transcription factors, Oct4, Sox2 and Rex1 in MSCs by RT-PCR. (C) Surface markers of MSCs were verified by the flow cytometry.

Supplementary Figure 5. Elevated blood pressure in offspring with different birth weight associated with sustained increased leptin levels.

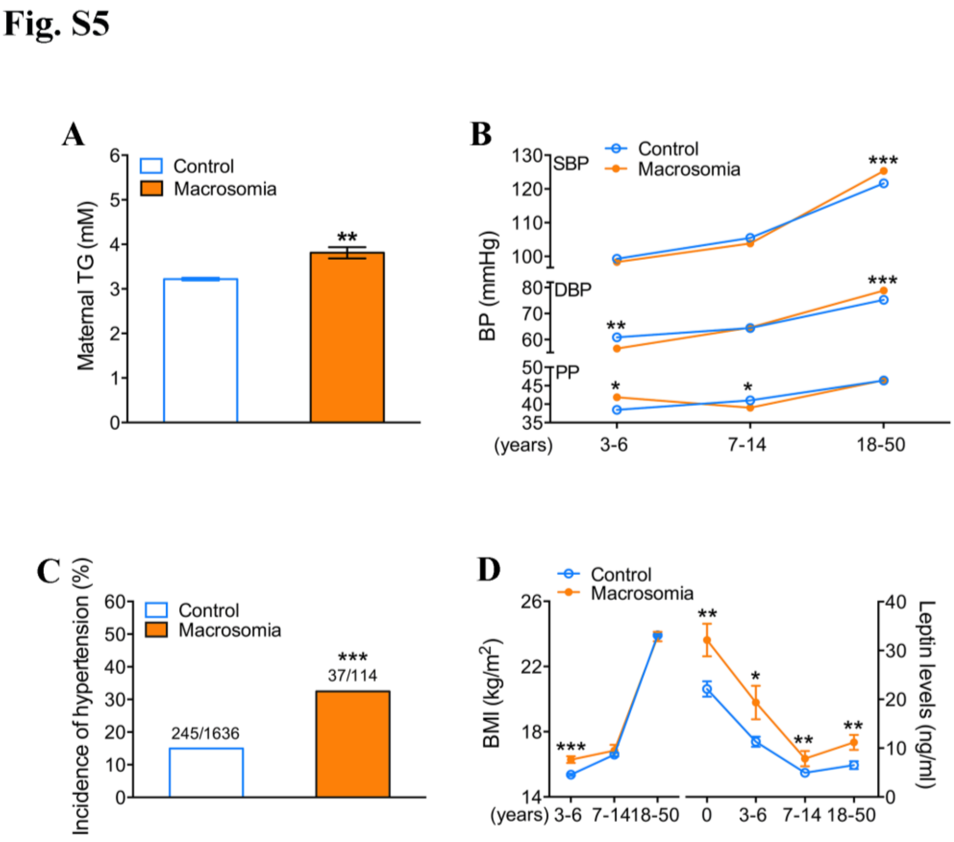

Fig. S5. Elevated blood pressure in offspring with different birth weight associated with sustained increased leptin levels. (A) Incidence of macrosomia in low (n=1625) and high (n=1062) mTG groups. (B) Blood pressures in offspring of different ages from macrosomia and control groups. (C) Incidence of hypertension in adults aged 30-50 years from macrosomia and control groups (SBP≥140 mmHg and/or DBP≥90 mmHg, OR 2.73, 95CI 1.80-4.13, p<0.0001). (D) BMI in offspring of different ages in control (n=776, 890 and 2892 for 3-6, 7-14 and 18-50 years, respectively) and macrosomia (n=82, 76 and 187 for 3-6, 7-14, 18-50 years, respectively) groups. Serum leptin levels in offspring of different ages in control (n=25, 19 and 12 for 3-6, 7-14 and 18-50 years, respectively) and macrosomia (n=15, 13 and 11 for 3-6, 7-14 and 18-50 years, respectively) groups. Values (in A, B and D are expressed as means ± SE, ***p<0.0001 **p<0.01, *p<0.05, compared to the corresponding control.

Original images of gels/blots for supplementary figure S2:

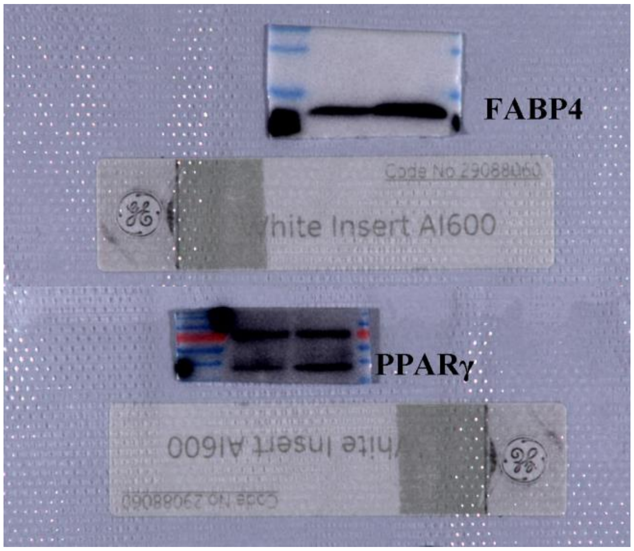

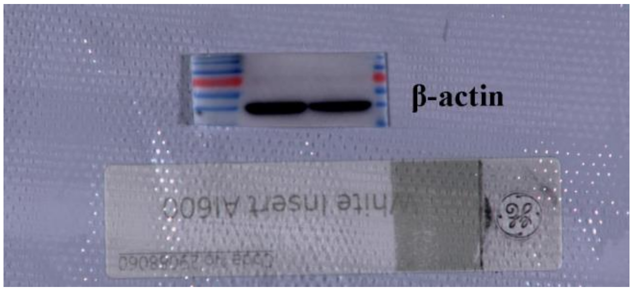

Original images of gels/blots for figure 4B and supplementary figure 3:

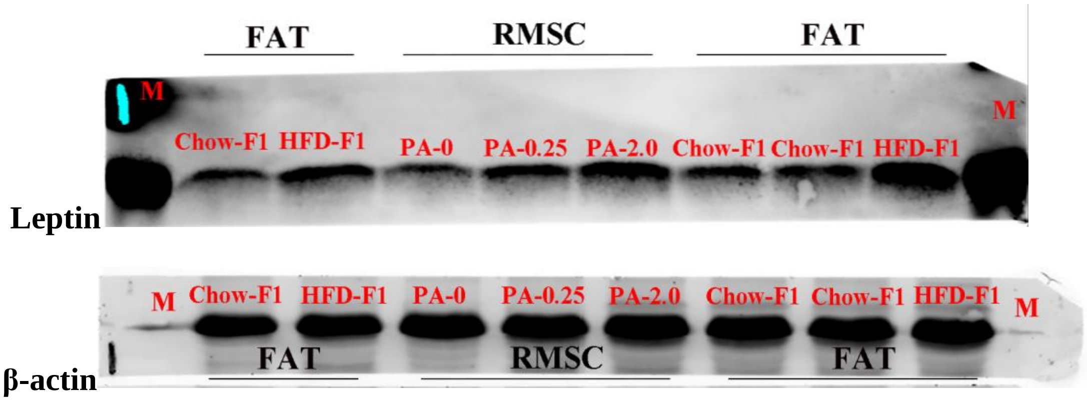

Supplementary Table S1. Maternal characteristics of newborns between different mTG groups.

	Low mTG (≤3.28mM, n=1625)	High mTG (>3.28mM, n=1062)	*P* value[1]
**Maternal**			
Age (year)	28.28±3.62[2]	28.10±13.64	0.270
Weight (kg)	69.00±7.78	69.57±8.05	0.071
Height (cm)	160.59±4.60	160.44±4.36	0.412
BMI of late pregnancy (kg/m^2)	26.75±2.81	27.02±2.92	0.019
Weight gain (kg)	16.51±3.89	16.67±3.99	0.329
Triglyceride (mM)	2.39±0.54	4.64±1.54	<0.0001
Total cholesterol (mM)	5.98±1.07	6.55±1.26	<0.0001
HDL cholesterol (mM)	1.88±0.41	1.75±0.40	<0.0001
LDL cholesterol (mM)	2.76±0.97	3.04±0.83	<0.0001
**Neonatal**			
Gestational age (wk)	38.90±1.01	38.94±1.09	0.490
Placenta area (cm^2)	363.88±82.94	361.58±76.82	0.838
Male/female sex (n)	853/772	558/504	1.000
Weight (g)	3388.96±397.64	3444.60±443.65	0.001
Length (cm)	50.77±1.02	50.84±1.07	0.676
Number of macrosomia (n)	144	168	<0.0001

Note: [1]Data were analyzed by using Student's t, Mann-Whitney U, and chi-square tests;

[2]Mean±SD (all such values).

Supplementary Table S2. Children's characteristics between different mTG groups

	Low mTG (≤3.28mM, n=59)	High mTG (>3.28mM, n=62)	*P* value[1]
Age (months)	58.72±13.12[2]	57.18±12.83	0.257
Birth weight (g)	3488.14±505.35	3733.87±591.52	0.008
Weight (kg)	19.29±3.41	18.87±3.97	0.265
Height (cm)	110.74±6.46	110.07±8.99	0.322
BMI (kg/m^2)	15.65±1.56	15.43±1.20	0.196
BMI z-score	-0.10±1.12	-0.18±0.89	0.330
Systolic BP (mmHg)	96.86±8.20	99.48±8.91	0.059
Diastolic BP (mmHg)	57.21±7.86	57.40±7.17	0.911
Mean arterial pressure (mmHg)	70.43±6.74	71.42±6.18	0.784
Pulse Pressure (mmHg)	39.65±9.01	42.08±10.09	0.096
Heart rate (times/minute)	96.11±14.01	97.47±12.72	0.303

Note: 1, Data were analyzed by using Student's t, Mann-Whitney U, and chi-square tests; 2, Mean±SD (all such values).

Supplementary Table S3. Maternal characteristics of newborns in normal birth weight (control) and high birth weight (macrosomia) groups.

	Control (2500-3999g, n=2375)	Macrosomia (≥4000g, n=312)	*P* value[1]
**Maternal**			
Age (year)	28.19±2.11[2]	28.24±1.77	0.866
Weight (kg)	68.52±4.37	75.00±4.69	<0.0001
Height (cm)	160.35±2.56	161.99±2.66	<0.0001
BMI of late pregnancy (kg/m^2)	26.64±1.59	28.58±1.76	<0.0001
Weight gain (kg)	16.43±2.22	17.7±2.51	<0.0001
Triglyceride (mM)	3.22±0.82	3.81±1.22	<0.0001
Total cholesterol (mM)	6.11±0.68	6.22±0.70	0.151
HDL cholesterol (mM)	1.85±0.24	1.71±0.24	<0.0001
LDL cholesterol (mM)	2.96±0.52	2.72±0.50	<0.0001
Fasting glucose (mM)	4.21±0.52	4.27±0.51	0.083
**Neonatal**			
Gestational age (wk)	38.87±0.60	39.35±0.56	<0.0001
Placenta area (cm^2)	320.28±28.78	382.00±50.25	<0.0001
Male/female sex (n)	1225/1150	199/113	<0.0001
Weight (g)	3317.28±190.67	4177.21±125.27	<0.0001
Length (cm)	49.90±0.32	51.00±0.62	<0.0001

Note: [1]Data were analyzed by using Student's t, Mann-Whitney U, and chisquare tests; [2]Mean±SD (all such values).

Supplementary Table S4. Anthropometric indexes and serum leptin in children aged 3-6 years.

	Control (2500-3999g, n=776)	Macrosomia (≥4000g, n=82)	*P* value[1]
Age（month）	50.18±10.31[2]	61.36±8.44	0.158
Male/female sex (n)	416/360	53/29	0.062
Height (cm)	104.71±7.52	108.12±6.62	<0.001
Weight (kg)	16.99±3.49	19.13±3.44	<0.001
BMI (kg/m^2)	15.36±1.82	16.29±1.87	<0.001
Systolic BP (mmHg)	99.32±10.74	98.35±7.50	0.306
Diastolic BP (mmHg)	60.87±8.94	56.51±6.51	0.003
Pulse Pressure (mmHg)	38.46±10.39	41.84±9.32	0.039

Note: [1]Data were analyzed by using Student's t, Mann-Whitney U, and chi-square tests; [2]Mean±SD (all such values); BP, blood pressure.

Supplementary Table S5. Anthropometric indexes and serum leptin in children aged 7-14 years.

	Control (2500-3999g, n=890)	Macrosomia (≥4000g, n=76)	*P* value[1]
Age (year)	8.98±1.88[2]	8.94±2.26	0.439
Male/female sex (n)	483/407	46/30	0.337
Height (cm)	134.57±12.73	138.78±12.97	0.003
Weight (kg)	30.63±9.92	33.13±10.97	0.018
BMI (kg/m^2)	16.60±3.37	16.84±3.06	0.269
Systolic BP (mmHg)	105.48±10.70	103.83±9.62	0.113
Diastolic BP (mmHg)	64.44±8.64	64.61±8.44	0.438
Pulse Pressure (mmHg)	41.03±9.67	39.02±6.73	0.048

Note: [1]Data were analyzed by using Student's t, Mann-Whitney U, and chi-square tests; [2]Mean±SD (all such values).

Supplementary Table S6. Anthropometric indexes and serum leptin in adults of 18-50 years old.

	Control (2500-3999g, n=2892)	Macrosomia (≥4000g, n=187)	*P* value[1]
Age (year)	31.02±6.69[2]	30.60±14.63	0.173
Male/female sex (n)	1609/1283	97/90	0.324
Height (cm)	167.11±7.55	169.21±7.95	<0.001
Weight (kg)	67.10±12.70	68.58±14.04	0.067
BMI (kg/m^2)	23.91±3.70	23.84±3.99	0.397
Systolic BP (mmHg)	121.66±13.88	125.32±17.04	<0.0001
Diastolic BP (mmHg)	75.26±10.01	78.84±12.50	<0.0001
Pulse Pressure (mmHg)	46.40±10.16	46.48±10.98	0.460

Note: [1]Data were analyzed by using Student's t, Mann-Whitney U, and chi-square tests; [2]Mean±SD (all such values).

Supplementary Table S7. Anthropometric indexes in adults of 30-50 years old.

	Control (2500-3999g, n=1636)	Macrosomia (≥4000g, n=114)	*P* value[1]
Age (year)	36.47±5.71[2]	36.08±5.70	0.245
Male/female sex (n)	761/875	54/60	0.923
Height (cm)	165.32±7.63	167.34±7.38	0.003
Weight (kg)	64.93±12.66	67.44±13.96	0.002
BMI (kg/m^2)	23.63±3.71	23.98±4.16	0.167
Systolic BP (mmHg)	122.45±14.64	128.22±18.65	<0.0001
Diastolic BP (mmHg)	76.40±10.65	80.74±14.19	<0.0001
Pulse Pressure (mmHg)	46.05±10.26	47.48±10.92	0.076

Note: [1]Data were analyzed by using Student's t, Mann-Whitney U, and chi-square tests; [2]Mean±SD (all such values).
